# Regional Difference in the Impact of COVID-19 Pandemic on Domain-Specific Physical Activity, Sedentary Behavior, Sleeping Time, and Step Count: Web-Based Cross-sectional Nationwide Survey and Accelerometer-Based Observational Study

**DOI:** 10.2196/39992

**Published:** 2023-02-20

**Authors:** Yosuke Yamada, Hideyuki Namba, Heiwa Date, Shinobu Kitayama, Yui Nakayama, Misaka Kimura, Hiroyuki Fujita, Motohiko Miyachi

**Affiliations:** 1 Department of Physical Activity Research National Institute of Biomedical Innovation, Health and Nutrition Osaka Japan; 2 Department of Liberal Arts and Science College of Science and Technology Nihon University Tokyo Japan; 3 Faculty of Data Science Shiga University Shiga Japan; 4 Department of Psychology University of Michigan Ann Arbor, MI United States; 5 Institute for Active Health Kyoto University of Advanced Science Kyoto Japan; 6 Faculty of Sport Sciences Waseda University Saitama Japan

**Keywords:** web-based survey, social distancing measure, transportation, physical activity record system, physical activity, sedentary, sleep, sleeping time, COVID-19, impact, pandemic, sleeping pattern, surveillance, demographic, regional, differences

## Abstract

**Background:**

Physical activity (PA) and sedentary behavior (SB) have been affected by the COVID-19 pandemic and its restrictive environments, such as social distancing and lockdown measures. However, regional differences in the changes in domain-specific PA and SB in response to the COVID-19 pandemic are not clearly understood.

**Objective:**

This study aimed to examine regional differences in domain-specific PA and SB, as well as sleeping time in response to the COVID-19 pandemic in Japan.

**Methods:**

A web-based cross-sectional nationwide survey and an accelerometer-based longitudinal observation were conducted. In the web-based survey, we recruited 150 Japanese men and 150 Japanese women for each of the following age groups: 20s, 30s, 40s, 50s, 60s, and 70s (n=1800). A total of 1627 adults provided valid responses to web-based surveillance from June to July 2020. Participants were recruited from urban (Greater Tokyo Area, n=1028), urban-rural (regional core cities, n=459), or rural (regional small and medium cities, n=140) areas. They answered sociodemographic and health-related questions and retrospectively registered the PA data of their average day before and during the COVID-19 pandemic in a web-based PA record system. In the accelerometer-based observation, PA and step count data were obtained using a triaxial accelerometer on people living in urban (n=370) and rural (n=308) areas.

**Results:**

Before the COVID-19 pandemic, there were no significant differences between these 3 regions in the time spent sleeping, staying at home, working or studying, and exercising (*P*>.05). By contrast, people living in urban areas had a longer duration of SB and transportation and a shorter duration of moderate-to-vigorous PA and lying or napping time compared with people living in rural areas (*P*>.05). During the COVID-19 pandemic, a significant decrease was observed in transportation time in urban (–7.2 min/day, *P*<.001) and urban-rural (–2.0 min/day, *P*=.009) areas but not in rural (–0.4 min/day, *P*=.52) areas. The moderate-to-vigorous PA was decreased in urban (–31.3 min/day, *P*<.001) and urban-rural (–30.0 min/day, *P*<.001) areas but not in rural areas (–17.3 min/day, *P*=.08). A significant increase was observed in time spent sleeping in urban (+22.4 min/day, *P*<.001) and urban-rural (+24.2 min/day, *P*<.001) but not in rural areas (+3.9 min/day, *P*=.74). Lying or napping was increased in urban (+14.9 min/day, *P*<.001) but not in rural areas (−6.9 min/day, *P*=.68). PA and step count obtained using an accelerometer significantly decreased in urban (*P*<.05) but not in rural areas (*P*>.05).

**Conclusions:**

The effect of the COVID-19 pandemic on PA and SB was significantly dependent on living area, even in a single country. The effects of PA and SB were greater in the Greater Tokyo Area and regional core cities but were not observed in regional small and medium cities in Japan.

## Introduction

Human lifestyles have changed in recent years, with an increasing number of people living a sedentary lifestyle, particularly in the countries with higher human development index [1]. Adequate amounts of physical activity (PA), limited sedentary behavior (SB), and proper sleeping time (ST) are essential for maintaining health [2-4]. Physical inactivity increases the risk of many adverse health conditions, such as coronary heart disease, type 2 diabetes, colon and breast cancers, mobility disabilities, and mortality [5].

The COVID-19 pandemic has changed the world and strongly affected the health of the people, with more than 640 million people with COVID-19, causing more than 6.6 million deaths worldwide [6]. Studies have indicated that the COVID-19 pandemic and its restrictive environment induced by the government’s statements about social distancing and lockdown measures affect PA, SB, and ST in children, adolescents, and young and older adults worldwide [7-15]. Stockwell et al [16] conducted a systematic review of the changes in PA and SB from before to during the COVID-19 pandemic restrictive environment. Most studies showed a decreased PA and an increased SB in the 66 included studies. They noted that different degrees of *lockdown* in different countries, even regions within a country, make it difficult to objectively assess how different degrees of restrictive environments impact behaviors. They also noted that most studies report PA without investigating in detail the types and durations of PA engaged before and during the lockdown. Thus, it would be beneficial to investigate these because the magnitude of changes will impact the effects on health.

For the Japanese population, a web-based survey using the short version of the International Physical Activity Questionnaire (IPAQ) found that PA decreased in April and recovered in June 2020 in older Japanese adults [7,8]. Another web-based survey using the Global Physical Activity Questionnaire (GPAQ) of Japanese workers found a significant increase in ST and a significant decrease in leisure-related moderate-to-vigorous physical activity (MVPA) during the COVID-19 outbreak (July 2020) compared to before the outbreak (February 2019) [13]. The IPAQ and GPAQ are considered world-standard questionnaires, with the advantage of comparing the results with worldwide data [17]. However, their external validity against the doubly labeled water (DLW) method is poor [18] compared with objective accelerometers [19,20]. The most recent meta-analysis also indicated that the criterion validity of IPAQ and GPAQ against objective smart trackers was low (weighted mean of *r*=0.23) [17]. Thus, more reliable systems to assess PA and SB in daily life are required for a stronger conclusion. In addition, it is essential to examine regional differences in PA and SB in response to the COVID-19 pandemic.

This study assessed a 24-hour domain-specific PA, SB, and ST in Japanese adults nationwide using a previously validated web-based simplified physical activity record (sPAR) system. In addition, we obtained longitudinal PA and step count data by a triaxial accelerometer on people living in urban and rural areas. We hypothesized that the impact of the COVID-19 pandemic and its restrictions (including government social distancing measures) on domain-specific PA, SB, ST, or step count vary by region of residence, even within a single country.

## Methods

### Data Source

The web-based surveillance was conducted through an internet research company (Cross Marketing Inc). Cross Marketing Inc and its partner companies have active panels with over 5 million people who registered their sociodemographic information in the company’s database and responded to at least one survey within the last year [21]. The company recruited 1800 adults from their active panels from June to July 2020; in total, 150 men and 150 women were recruited for each of the following age groups: 20s, 30s, 40s, 50s, 60s, and 70s. Individuals completed the web-based sPAR system, which was included before and during the COVID-19 pandemic. The company provided the URL via email and requested each participant to complete a survey. All the participants signed a web-based informed consent form. The research company removed participants’ names, addresses, contact information, and any other details that might be used to identify individuals prior to transferring the data to researchers from the data. A total of 1627 adults provided valid responses for this web-based survey. Self-reported age, height, and weight data were obtained. The participants were categorized by gender and residential area, which were divided into urban (Greater Tokyo Area), urban-rural (regional core cities), and rural (regional small and medium cities), based on the Fourth National Comprehensive Development Plan, Japanese National Land Agency [22].

### Ethical Considerations

This study was conducted with approval from the ethics committees of Kyoto University of Advanced Science (KUAS no.20-3) and the National Institutes of Biomedical Innovation, Health and Nutrition (NIBIOHN no. 202). Participants were provided with an information sheet on the landing page of the web-based survey. Participants were only allowed to continue participating if they acknowledged that they had read the information sheet and agreed to give informed consent.

### Web-Based PA Record

The sPAR is a unique system for assessing 24-hour domain-specific PA, SB, and ST [23]. The external validity is moderate to high (*r*=0.8 for total energy expenditure and *r*=0.63 for PA level) [23-25]. One study developed a web-based sPAR system and found that external and criterion validity were also moderate to high (*r*=0.68 for activity energy expenditure [AEE] measured by DLW and *r*=0.87 for total energy expenditure; *r*=0.77 for AEE; *r*=0.71 for PA level assessed by a validated triaxial accelerometer) [26,27]. The external validity from another study of a triaxial accelerometer and sPAR against the DLW method was similar and high [24].

**Figure 1:**
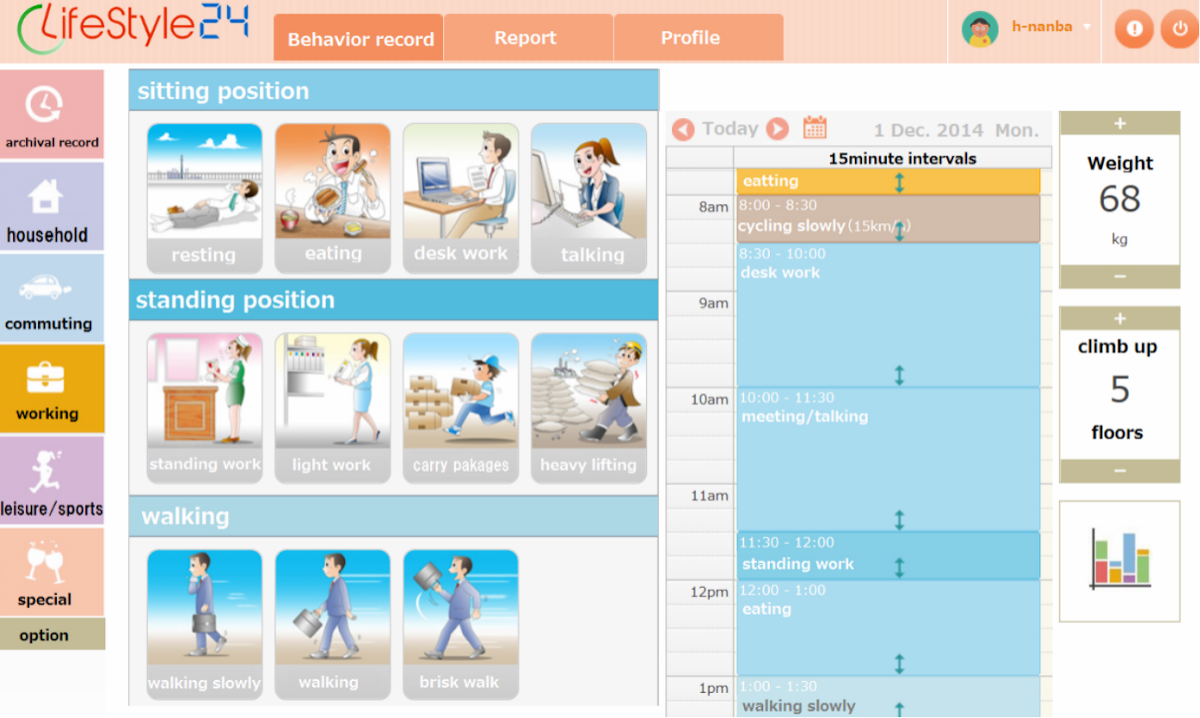
Screenshot of the web-based simplified physical activity record (sPAR) system (Namba et al [28]).

A representative screenshot of the web-based sPAR system is shown in Figure 1. The system collected recalled activities over 24 hours. The participants were instructed to respond to questions about a typical day that is not a weekend or holiday. The participants selected one of the preset activities performed in each of the four categories (household, transportation, work-related, and leisure or sports) for 15-minute intervals. In this system, 91 activities among the four categories were preset. The details of the web-based sPAR system have been previously described [26,28]. The intensity of each activity was entered into the system as metabolic equivalent of tasks (METs) based on the 2011 Compendium of Physical Activities [29]. MVPA (≥3.0 METs) and SB (1.0-1.5 METs) were calculated based on the MET value of each activity.

### Accelerometer

In addition to this web-based survey, we also used the data set of a triaxial accelerometer that had been previously published as a short research paper [11]. The original data contained 628,111 observation days from January 1, 2019, to January 1, 2021, from 1167 unique users of the accelerometer nationwide in Japan. From the 1167 unique users’ data, people who lived in the Greater Tokyo Area (n=370) and those who lived in rural areas (n=308) were selected to compare urban and rural differences in this study based on the information about the prefecture in which they lived [11]. The average step counts and AEEs for January 2019, April 2019, January 2020, and April 2020 were calculated. Step counts and AEE were monitored using a triaxial accelerometer (EW-NK63; Panasonic). This accelerometer was manufactured based on the Actimarker (EW4800, Panasonic), an accelerometer for research use, as a low-cost version of the one for the general public [30]. Daily step counts and AEE were stored on a server via participants’ smartphones. All participants read the explanation of the study and indicated their understanding before providing informed consent. The details of the METs calculations have been previously described [31,32]. The output of the accelerometer was highly correlated (*R*^2^=0.86) with the METs while walking or running at 7 speeds ranging from 40 to 160 m/min^−1^ and during the following 7 daily activities: performing self-care while standing, changing clothes, cooking, simulating eating supper, washing dishes, doing laundry, and using a vacuum cleaner [31,32]. AEE was calculated from 24-hour average METs, and this accelerometer highly correlates with the DLW method [20,24].

### Statistical Analyses

Data are shown as means and standard deviations in the tables and as means and standard errors in the figures. One-way ANOVA was performed to compare age, and a chi-square test was conducted to compare the percentage of either gender between residential areas. Socioeconomic status, smoking, alcohol consumption, and health-related information, including medical history, were extracted from the web-based survey. Psychological distress was assessed by the Kessler Psychological Distress Scale (K6) screening scales [33-35]. One-way analysis of covariance was performed to compare other values between residential areas with the variables in Tables 1 and 2. A 2-tailed paired *t* test was conducted to compare values before and during the COVID-19 pandemic. The results were considered significant at a *P*<.05. SPSS version 22 (IBM Corp) was used for all statistical analyses.

**Table 1:** Physical characteristics of the participants (n=1627).

Characteristics	Urban (n=1028)	Urban-rural (n=459)	Rural (n=140)	*P* value
Men, n (%)	542 (52.7)	267 (58.2)	79 (56.4)	.21
Age (years), mean (SD)	49 (17)	48 (16)	46 (15)	.20
Height (cm), mean (SD)	164 (8)	165 (11)	164 (9)	.77
Weight (kg), mean (SD)	61 (20)	61 (13)	62 (14)	.68

**Table 2:** Social and health status of the participants (N=1627).

Social and health status	Urban (n=1028), n (%)	Urban-rural (n=459), n (%)	Rural (n=140), n (%)	*P* value
Current worker or student	701 (68.2)	315 (68.6)	108 (77.1)	.096
Alcohol drinker	609 (59.2)	250 (54.5)	53 (37.9)	*<.001*
Current smoker	210 (20.4)	92 (20.0)	35 (25.0)	.42
Living alone	191 (18.6)	73 (15.9)	20 (14.3)	.27
Living with pets	220 (21.4)	119 (25.9)	39 (27.9)	.07
Household income ≥10 million yen (≥US $77,000)	117 (11.4)	28 (6.1)	10 (7.1)	*.004*
Good subjective economic status	541 (52.6)	183 (39.9)	62 (44.3)	*<.001*
Education ≥13 years	821 (79.9)	296 (64.5)	83 (59.3)	*<.001*
Good subjective health	836 (81.3)	350 (76.3)	107 (76.4)	.05
Psychological Distress (K6^a^ score≥5)	433 (42.1)	221 (48.1)	62 (44.3)	.096
Having purpose in life	725 (70.5)	310 (67.5)	96 (68.6)	.496
No medication	694 (67.5)	293 (63.8)	93 (66.4)	.38
Hypertension	158 (15.4)	82 (17.9)	14 (10.0)	.08
Hyperlipidemia	74 (7.2)	27 (5.9)	5 (3.6)	.21
Diabetes	52 (5.1)	24 (5.2)	5 (3.6)	.72
Heart disease	17 (1.7)	10 (2.2)	5 (3.6)	.29
Cancer	16 (1.6)	9 (2.0)	1 (0.7)	.58
Depression	39 (3.8)	26 (5.7)	9 (6.4)	.15

^a^K6: Kessler Psychological Distress Scale.

## Results

The physical characteristics of the participants are shown in Table 1. No significant differences were observed in age, gender, height, or weight among the 3 residential area categories (*P*>.05). Table 2 shows the social and health status of the participants. The urban area had a higher percentage of alcohol drinkers, people with higher household income and subjective economic status, and those with higher education than other areas (*P*<.05). However, other variables were not significantly different between areas (*P*>.05).

In Table 3, Before the COVID-19 pandemic, there were no significant differences in sleeping time, time spent at home, working or studying time, and exercise time (*P*>.05) in the 3 regions. By contrast, people living in urban areas had a longer duration of SB and transportation and a shorter duration of MVPA and lying or napping time compared with people living in rural areas (*P*<.05). Significant and negative correlation was observed between work duration and ST (*r*=‒0.373, *P*<.001), and between transportation time and ST (*r*=‒0.289, *P*<.001).

**Figure 2:**
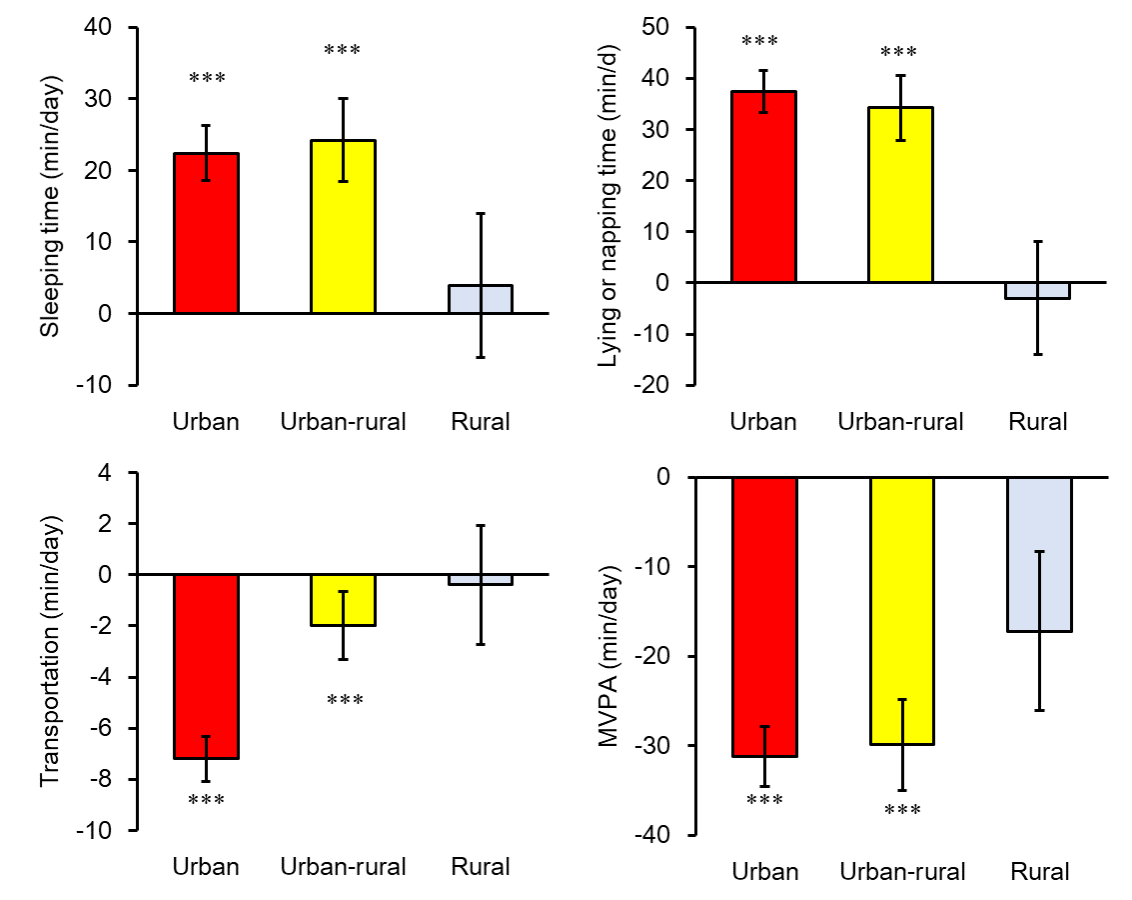
The changes in sleeping time, sedentary behavior, and physical activity from before to during the COVID-19 pandemic were assessed by a web-based physical activity record system (mean and SE). Significant differences were observed between the urban (Greater Tokyo Area) or urban-rural areas and the rural area in changes in the duration of sleeping, lying or napping, transportation, and moderate-to-vigorous physical activity (MVPA). ***Signficantly changed from before the COVID-19 pandemic (*P*<.001).

Table 4 and Figure 2 show changes in ST, SB, transportation, and MVPA before and during the COVID-19 pandemic. A significant decrease was observed in transportation time (−7.2 min/day, *P*<.001) and urban-rural (−2.0 min/day, *P*=.009) areas but not in rural (−0.4 min/day, *P*=.52) areas during the pandemic. The MVPA was decreased in urban (−31.3 min/day, *P*<.001) and urban-rural (−30.0 min/day, *P*<.001) areas but not in rural areas (−17.3 min/day, *P*=.08). A significant increase was observed in time spent sleeping in urban (+22.4 min/day, *P*<.001) and urban-rural (+24.2 min/day, *P*<.001) but not in rural areas (+3.9 min/day, *P*=.74). Lying or napping was increased in urban (+14.9 min/day, *P*<.001) but not in rural areas (−6.9 min/day, *P*=.68).

**Figure 3:**
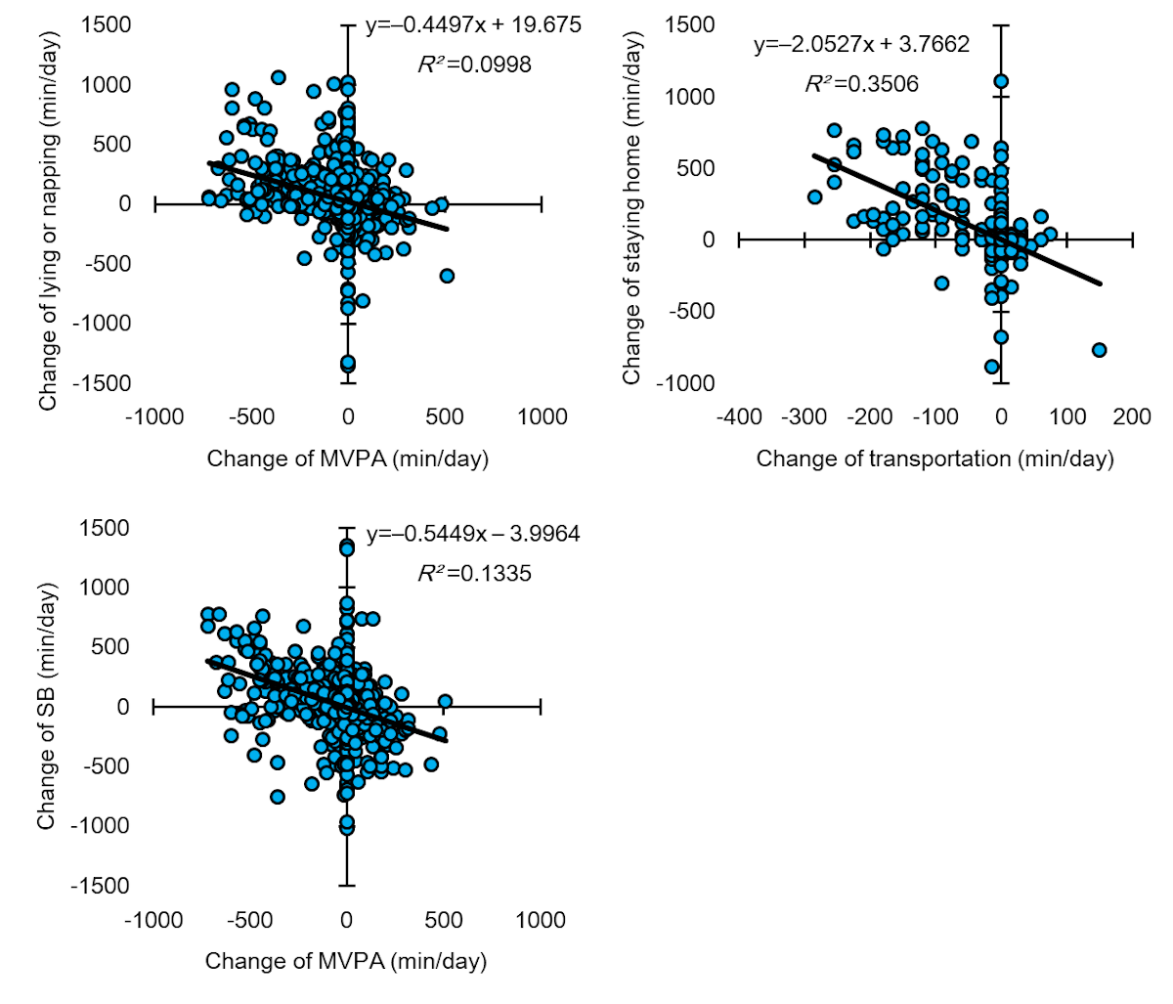
The relationship between changes in physical activity and sedentary behavior (SB) during the COVID-19 pandemic. The change in moderate-to-vigorous physical activity (MVPA) duration was significantly and negatively correlated with the change in the duration of lying or napping and SB. The change in transport duration was significantly and negatively correlated with the change in the duration of staying home.

Figure 3 shows the relationship between the changes in PA and SB during the COVID-19 pandemic. The change in the duration of transportation was significantly and negatively correlated with the change in the duration of staying at home (*P*<.05). The change in MVPA duration was significantly and negatively correlated with the change in the duration of lying or napping and the change of the duration of SB (*P*<.05).

**Figure 4:**
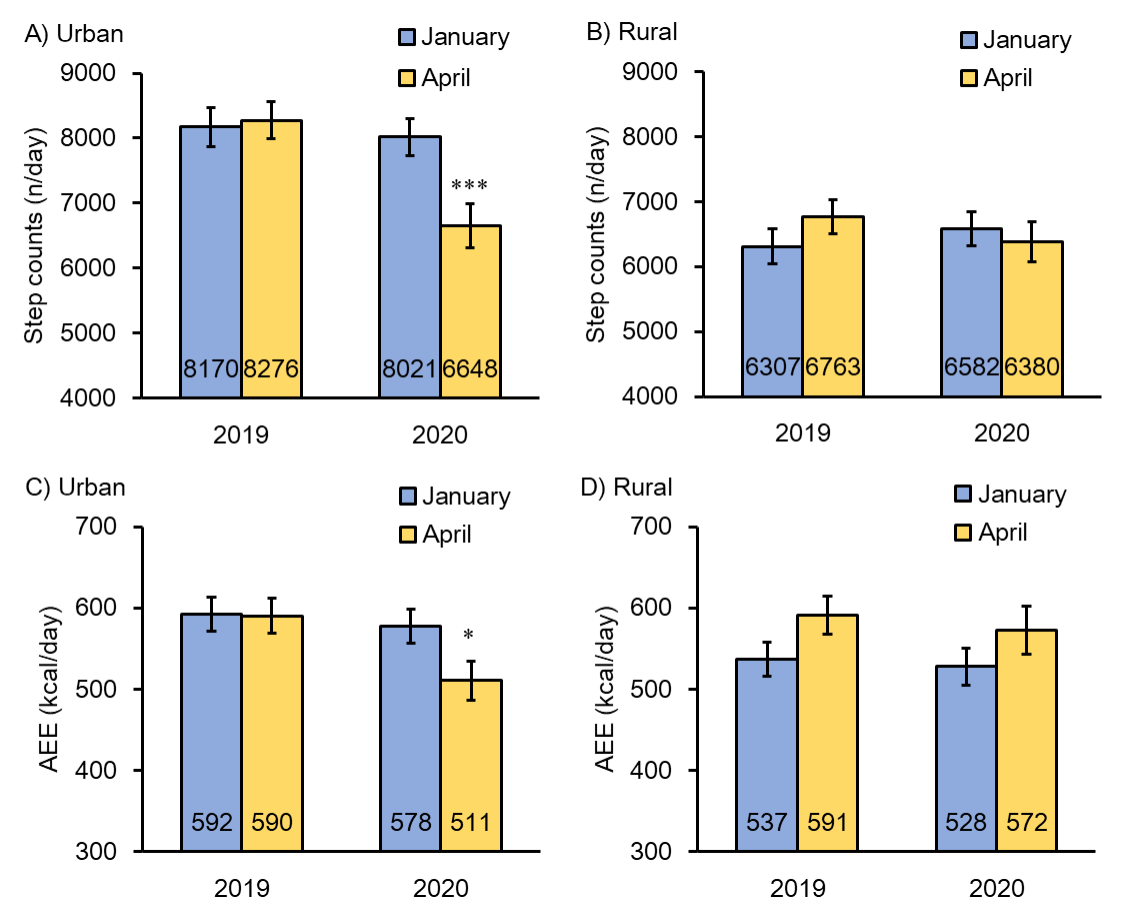
Objectively measured step counts and activity energy expenditure (AEE) by triaxial accelerometer (mean and SE). There is no significant difference between January and April 2019 in step counts and AEE in urban and rural prefectures. The step counts (*P*<.001) and AEE (*P*<.05) in April 2020 were significantly decreased in urban prefectures when January and April 2019 are compared to January 2020.

Figure 4 shows the results of the accelerometer monitoring. The step counts and AEE obtained by an accelerometer significantly decreased during the COVID-19 pandemic in urban areas (*P*<.05) but not in rural areas (*P*>.05).

**Table 3:** Physical activities and sedentary behavior of the participants (n=1627).

Variables		Urban (n=1028), mean (SD)	Urban-rural, (n=459), mean (SD)	Rural, (n=140), mean (SD)	*P* value	
**Before the COVID-19 pandemic**						
	Sleeping time (min/day)	453 (139)	462 (145)	460 (137)	.51	
	Lying or napping time (min/day)	99 (153)	111 (151)	132 (194)	*.045*	
	SB^a^ (min/day)	610 (248)	592 (242)	528 (252)	*.001*	
	MVPA^b^ (min/day)	168 (181)	169 (185)	214 (212)	*.02*	
	Staying time at home (min/day)	639 (301)	650 (284)	613 (275)	.41	
	Transportation (min/day)	65 (85)	48 (72)	41 (49)	*<.001*	
	Working or studying time (min/day)	265 (262)	266 (260)	309 (258)	.17	
	Exercise time (min/day)	10 (30)	6 (21)	10 (62)	.07	
**During the COVID-19 pandemic**						
	Sleeping time (min/day)	476 (165)^c^	487 (181)^c^	464 (141)	.32	
	Lying or napping time (min/day)	114 (179)^c^	121 (175)	124 (208)	.68	
	SB (min/day)	625 (259)^d^	595 (251)	551 (273)	*.002*	
	MVPA (min/day)	137 (161)^c^	139 (168)^c^	197 (205)	*<.001*	
	Staying time at home (min/day)	658 (299)^c^	654 (286)	630 (279)	.57	
	Transportation (min/day)	57 (83)^c^	46 (72)^d^	41 (49)	*.007*	
	Working or studying time (min/day)	249 (261)^c^	260 (258)^e^	296 (261)	.12	
	Exercise time (min/day)	10 (29)	5 (19)	10 (62)	*.03*	

^a^SB: sedentary behavior.

^b^MVPA: moderate-to-vigorous physical activity.

^c^Significant difference versus before the COVID-19 pandemic: *P*<.001.

^d^Significant difference versus before the COVID-19 pandemic: *P*<.01.

^e^Significant difference versus before the COVID-19 pandemic: *P*<.05.

**Table 4:** Change in score of physical activity and sedentary behavior in the participants (N=1928).

Variable	Urban (n=1028), mean (IQR)	*P* value	Urban-rural (n=459), mean (IQR)	*P* value	Rural (n=140), mean (IQR)	*P* value
Sleeping time (min/day)	22.4 (14.8, 30.0)	*<.001*	24.2 (11, 37.4)	*<.001*	3.9 (–19, 26.7)	.74
Lying or napping time (min/day)	14.9 (6.2, 23.7)	*.001*	10.0 (–2.7, 22.7)	.12	–6.9 (–40.1, 26.4)	.68
SB^a^ (min/day)	14.9 (4.1, 25.8)	*.007*	2.9 (–14.6, 20.3)	.75	22.5 (–7.5, 52.5)	.14
MVPA^b^ (min/day)	–31.3 (–38.8, –23.7)	*<.001*	–30.0 (–40.8, –19.1)	*<.001*	–17.3 (–36.3, 1.8)	.08
Staying time at home (min/day)	18.8 (12.1, 25.4)	*<.001*	3.7 (–3.6, 11.0)	.33	17.3 (–4.0, 38.5)	.11
Transportation (min/day)	–7.2 (-9.4, -5.0)	*<.001*	–2.0 (–3.5, –0.5)	*.009*	–0.4 (–1.7, 0.9)	.52
Working or studying time (min/day)	–16.3 (–21.7, –11)	*<.001*	–5.6 (–10.1, –1.2)	*.01*	–13.4 (–28.7, 1.9)	.09
Exercise time (min/day)	0.0 (–0.9, 0.9)	.98	–0.6 (–1.3, 0.1)	.09	–0.2 (–0.7, 0.3)	.42

^a^SB: sedentary behavior.

^b^MVPA: moderate-to-vigorous physical activity.

## Discussion

### Principal Findings

This study clearly shows regional differences in domain-specific PA, SB, and ST changes in response to the COVID-19 pandemic. The Japanese government first declared a state of emergency in response to the COVID-19 outbreak on April 7, 2020 [36,37]. The effects of the COVID-19 outbreak and the government-declared state of emergency were significantly larger in the urban (Greater Tokyo Area) and urban-rural (regional core cities) areas than in rural areas (regional small and medium cities). Particularly, MVPA decreased by 31 min/day (–18.4%), from 168 min/day before to 137 min/day during the COVID-19 pandemic. Sleeping time (+26 min/day on average) and lying or napping time (+36 min/day on average) increased significantly during the COVID-19 pandemic in the urban and urban-rural areas. In addition, the duration of transportation was significantly decreased by 7 min/day, from 65 min/day before to 57 min/day during the COVID-19 pandemic in the urban area. In addition, we confirmed these regional differences using the triaxial accelerometer as an objective method.

Several previous studies have observed similar patterns in other population groups [7,13,38,39]. However, many surveys that addressed domain-specific PA used PA questionnaires, such as the IPAQ and GPAQ. The external and criterion validity of the PA questionnaires was relatively low when compared with a gold standard method, such as the DLW method, or with objective accelerometers [17-20]. Thus, more reliable systems to assess PA and SB in daily life are required for a stronger conclusion. The web-based sPAR system is a unique method for obtaining PA and SB data with moderate or high external validity [23,26,27].

In this study, Japanese living in urban or urban-rural areas were found to have spent more time lying and sleeping during the COVID-19 pandemic than before the pandemic. The average ST of the participants was 455 min/day before the COVID-19 pandemic. This is consistent with the Organization for Economic Co-operation and Development (OECD) statistics in 2019, which shows 442 min/day as the average sleep duration for individuals in Japan [40]. Japan has the shortest ST compared to other countries [40]. OECD Better Life Index stated that “the percentage of employees working very long hours is higher in Japan than the OECD average of 11%, and full-time workers devote less of their day to personal care and leisure than the OECD average” [41]. This survey found a significant and negative correlation between working duration and ST (*P*<.05).

In Japan, several companies have introduced and supported some level of teleworking to avoid denseness in response to government statements [18]. Our study found a significant and negative correlation between transportation time and ST. The average transportation time in the Greater Tokyo Area was 65 min/day, which was significantly longer than that in the rural areas. Teleworking leads to less time traveling for work, and people might sleep more than they did before the COVID-19 pandemic. This might be a favorable effect of teleworking. However, people have spent more time in a lying position during the COVID-19 pandemic. A recent study found that an increase in SB was significantly associated with an increase in the motivation and PA aspects of fatigue [18]. Therefore, people who live in urban or urban-rural areas and spend more SB time might experience increased fatigue. However, further research is required [42].

MVPA is defined as any activity ≥3 METs, including sports, exercise, and nonexercise PA, such as walking or bicycling to supermarkets or active periods of childcare. In this study, significantly decreased exercise time was not observed in all areas (*P*>.05); however, MVPA duration significantly and largely decreased during the COVID-19 pandemic (*P*<.001). Our study indicated that nonexercise MVPA decreased during the COVID-19 pandemic by about 30 min/day in the urban and urban-rural areas but not in rural areas in Japan.

One of the factors contributing to the regional differences is the difference in population density. The Tokyo metropolitan area has a very high population density. Thus, requests for self-restraint from the government were more severe than in smaller regional cities. It is also likely that a stronger response was seen in terms of people’s behavior to avoid crowds. Another factor that may have contributed to the regional differences is the occupational characteristics of the residents. In rural small and medium-sized cities, a smaller percentage of residents are office workers, university faculty, and people from other professional occupations, while a higher percentage comprise blue-collar workers and agricultural and workers in the fishing industry. We believe that differences in population density in the places where they work or live and whether they can be replaced by teleworking also had an impact on the change of PA and SB.

### Limitations

Our study has some methodological limitations. First, our web-based survey and accelerometer-based study used convenient samples without random sampling; thus, we did not calculate sample sizes and effect sizes in this study. Those not using the internet or accelerometers, especially older people, could not be included in this study. Therefore, this study’s results do not reflect the Japanese population characteristics; participants could be more health conscious than the general population, and our study potentially includes selection bias. Second, possible inconsistency of PA levels on workdays and weekends may exist. That is, the 24-hour sPAR data from a single acquisition may not represent a typical week for the PA level of the study participants. Particularly, the current system cannot examine weekly or monthly exercise habits. In addition, while we obtained the physical and mental health status in the web-based survey, we could not obtain the physical and mental health status in the accelerometer study, which may affect the results. Finally, as the main limitation of this study is based on the study design, it cannot be argued that such differences are based on the COVID-19 pandemic or the aging effect.

### Conclusion

This research, using a web-based sPAR system, found several lifestyle changes, indicating that people became less active and spent a longer amount of time in the lying position in urban and urban-rural areas during the COVID-19 pandemic. In addition, we found that Japanese living in urban and urban-rural areas had increased sleeping time during the COVID-19 pandemic. Longer sleep duration during the COVID-19 pandemic may favor the population’s health, as Japan has the shortest sleeping time worldwide. However, the less time spent on MVPA and the longer time spent on SB, particularly in the lying position, would have an unfavorable effect on health. The sociopsychological aspects of the effect of such lifestyle change should be examined in future studies.

## Abbreviations

AEE: activity energy expenditure
DLW: doubly labeled water
GPAQ: Global Physical Activity Questionnaire
IPAQ: International Physical Activity Questionnaire
MET: metabolic equivalent of task
MVPA: moderate-to-vigorous physical activity
OECD: Organization for Economic Co-operation and Development
PA: physical activity
SB: sedentary behavior
sPAR: simplified physical activity record
ST: sleeping time
